# Classic and Golli Myelin Basic Protein have distinct developmental trajectories in human visual cortex

**DOI:** 10.3389/fnins.2015.00138

**Published:** 2015-04-24

**Authors:** Caitlin R. Siu, Justin L. Balsor, David G. Jones, Kathryn M. Murphy

**Affiliations:** ^1^McMaster Integrative Neuroscience Discovery and Study Program, McMaster UniversityHamilton, ON, Canada; ^2^Pairwise Affinity Inc.Dundas, ON, Canada; ^3^Psychology, Neuroscience and Behaviour, McMaster UniversityHamilton, ON, Canada

**Keywords:** MBP, human, myelin, development, aging, western blotting, golli, visual cortex

## Abstract

Traditionally, myelin is viewed as insulation around axons, however, more recent studies have shown it also plays an important role in plasticity, axonal metabolism, and neuroimmune signaling. Myelin is a complex multi-protein structure composed of hundreds of proteins, with Myelin Basic Protein (MBP) being the most studied. MBP has two families: Classic-MBP that is necessary for activity driven compaction of myelin around axons, and Golli-MBP that is found in neurons, oligodendrocytes, and T-cells. Furthermore, Golli-MBP has been called a “molecular link” between the nervous and immune systems. In visual cortex specifically, myelin proteins interact with immune processes to affect experience-dependent plasticity. We studied myelin in human visual cortex using Western blotting to quantify Classic- and Golli-MBP expression in post-mortem tissue samples ranging in age from 20 days to 80 years. We found that Classic- and Golli-MBP have different patterns of change across the lifespan. Classic-MBP gradually increases to 42 years and then declines into aging. Golli-MBP has early developmental changes that are coincident with milestones in visual system sensitive period, and gradually increases into aging. There are three stages in the balance between Classic- and Golli-MBP expression, with Golli-MBP dominating early, then shifting to Classic-MBP, and back to Golli-MBP in aging. Also Golli-MBP has a wave of high inter-individual variability during childhood. These results about cortical MBP expression are timely because they compliment recent advances in MRI techniques that produce high resolution maps of cortical myelin in normal and diseased brain. In addition, the unique pattern of Golli-MBP expression across the lifespan suggests that it supports high levels of neuroimmune interaction in cortical development and in aging.

## Introduction

The last decade has seen renewed interest in studying myelin in the cortex because it is involved in neuroplasticity, neurodegeneration, and neuroinflammation. As maturation of cortical myelin limits developmental plasticity (McGee et al., 2005), changes in aging are linked with cognitive decline (Peters et al., 2008), and myelin abnormalities contribute to neurodegenerative and neuropsychiatric disease (Roussos and Haroutunian, 2014; Mighdoll et al., 2015). Novel brain imaging techniques are producing high resolution maps of myelin in human cortex (Glasser and van Essen, 2011; Grydeland et al., 2013; Shafee et al., 2015) that can follow these changes in cortical myelin during adaptive or maladaptive plasticity. Myelin, however, is a complex multi-protein structure and we need to know more about the expression of proteins that make up myelin in the human cortex to link these new brain imaging techniques with disease-related changes in myelin proteins.

In the cortex, oligodendrocytes are the second most abundant type of neuroglial cell and they form myelin. Myelin is made up of hundreds of proteins, many of which have multiple families and isoforms. The most commonly used marker for myelin expression is Myelin Basic Protein (MBP). MBP makes up about 30% of all myelin proteins and is composed of two families, Classic- and Golli-MBP, each with multiple isoforms and post-translational modifications (Pribyl et al., 1996; Harauz et al., 2009; Harauz and Boggs, 2013) that individually contribute to myelination (Jacobs et al., 2005, 2009; Harauz et al., 2009). Classic-MBP isoforms (18.5–21.5 kDa in human) are found in mature oligodendrocytes and myelin sheaths, and have a key role in activity driven compaction of myelin around axons (Wake et al., 2011). Expression of Classic-MBP increases during cortical development (Miller et al., 2012) and immunohistochemical labeling of Classic-MBP is correlated with traditional myelin staining. In contrast, Golli-MBP isoforms (33–35 kDa) are found in early developing oligodendrocytes, neurons and immune cells, with functions that extend beyond the myelin sheath and include regulating oligodendrocyte proliferation and migration (Paez et al., 2011). Golli-MBP is highly expressed prenatally in neurons and oligodendrocytes, even before the process of myelination begins (Tosic et al., 2002) and has been called a “molecular link” between the nervous and immune systems (Pribyl et al., 1993).

The primary sensory cortices are heavily myelinated (Geyer et al., 2011) and are good cortical regions to study developmental changes in expression of both families of MBP. Expression of MBP and myelination in the visual cortex is affected by neuropsychiatric disease and genetic manipulation of Golli-MBP. In schizophrenia, there is reduced MBP mRNA in human visual cortex, as well as other cortical areas, suggesting that MBP expression in visual cortex is vulnerable to neuropsychiatric disease (Matthews et al., 2012). Furthermore, abnormalities in genes that code for Golli-MBP are linked with significant risk for schizophrenia (Baruch et al., 2009). Although Golli-MBP is not part of the myelin sheath, it is necessary for normal development of myelination (Jacobs et al., 2005). Golli-MBP knockout (KO) mice have an abnormal balance between Golli- and Classic-MBP that leads to profound hypomyelination restricted to the visual cortex (Jacobs et al., 2005). While Golli-MBP overexpressing mice have delayed development of myelination (Jacobs et al., 2009). Several lines of evidence have linked myelin (McGee et al., 2005) and neuroimmune signaling (Syken et al., 2006) with the end of the sensitive period in the visual cortex. Since Golli-MBP has been called a “molecular link” between the nervous and immune systems (Pribyl et al., 1993) it is timely to characterize its expression in human visual cortex.

To address how the two families of MBP proteins change across the lifespan, we used Western blotting to quantify expression of Classic- and Golli-MBP in post-mortem tissue samples from human primary visual cortex. We used model fitting to characterize developmental trajectories that capture how Classic- and Golli-MBP change across the lifespan. We analyzed changes in the relative expression of Classic- and Golli-MBP to determine how the balance between these families of MBP vary in human visual cortex. Finally, we compared inter-individual variability in expression of Classic- and Golli-MBP to determine if there are times in development with higher inter-individual variability.

## Materials and methods

### Samples and tissue

Tissue samples from human primary visual cortex were obtained from the Brain and Tissue Bank for Developmental Disorders at the University of Maryland (Baltimore, MD, USA) and were approved for use by the McMaster University Research Ethics Board. Samples were taken from the posterior pole of the left hemisphere of human visual cortex, and included both superior and inferior portions of the calcarine fissure, according to the gyral and sulcal landmarks. Cortical samples were obtained from individuals with no history of mental health or neurological disorders, and all causes of death were natural, or with minimal trauma. Samples were obtained within 23 h post-mortem, and were rapidly frozen at the Brain and Tissue Bank after being sectioned coronally in 1-cm intervals, rinsed with water, blotted dry, placed in a quick-freeze bath (dry ice and isopentane), and stored frozen (−80 °C). A total of 31 cases were used in this study, ranging in age from 20 days to 79 years (Table 1).

**Table 1:** **Cases used in the study**.

**Age group**	**Age**	**Post-mortem interval (H)**	**Sex**
Neonatal	20 days	9	M
Neonatal	20 days	14	F
Neonatal	86 days	23	F
Neonatal	96 days	12	M
Neonatal	98 days	16	M
Neonatal	119 days	22	M
Neonatal	120 days	23	M
Infant	133 days	16	M
Infant	136 days	11	F
Infant	273 days	10	M
Young Children	1 year 123 days	21	M
Young Children	2 years 57 days	21	F
Young Children	2 years 75 days	11	F
Young Children	3 years 123 days	11	F
Young Children	4 years 203 days	15	M
Young Children	4 years 258 days	17	M
Older Children	5 years 144 days	17	M
Older Children	8 years 50 days	20	F
Older Children	8 years 214 days	20	F
Older Children	9 years 46 days	20	F
Teens	12 years 164 days	22	M
Teens	13 years 99 days	5	M
Teens	15 years 81 days	16	M
Teens	19 years 76 days	16	F
Young Adults	22 years 359 days	4	M
Young Adults	32 years 223 days	13	M
Young Adults	50 years 156 days	8	M
Young Adults	53 years 330 days	5	F
Older Adults	69 years 110 days	12	M
Older Adults	71 years 333 days	9	F
Older Adults	79 years 181 days	14	F

### Tissue-sample preparation

Small pieces of tissue samples (75–150 mg) were cut from 1-cm thick coronal slices of primary visual cortex (area V1), and suspended in cold homogenization buffer (1 ml buffer:50 mg tissue; 0.5 mM DTT, 1 mM EDTA, 2 mM EGTA, 10 mM HEPES, 10 mg/L leupeptin, 100 nM microcystin, 0.1 mM PMSF, 50 mg/L soybean trypsin inhibitor). Samples were homogenized using the FastPrep®−24 Tissue and Cell Homogenizer (MP Biomedicals, Solon, OH, USA) by placing the piece of tissue and buffer in a lysing matrix D homogenization tube (MP Biomedicals, Solon, OH, USA) and homogenizing for 40 s at 6/ms. After homogenization, sodium-dodecyl-sulfate 10% (SDS) was added to each sample to further unravel proteins in preparation for gel electrophoresis. Total protein concentrations were determined using a bicinchonic acid (BCA) assay (Pierce, Rockford, IL, USA). A control sample was made by combining a small amount of the homogenized tissue sample from each case.

### Immunoblotting

Homogenized tissue samples (20 μg) were separated on SDS polyacrylamide gels (SDS-PAGE) and transferred to polyvinylidene difluoride (PVDF-FL) membranes (Millipore, Billerica, MA, USA) using electroblotting in BupH Tris Glycine Transfer Buffer (Thermo Scientific, Waltham, MA, USA). Each sample was run 3 times. Each blot was loaded with a protein standards ladder and a control sample. Blots were pre-incubated for 1 h in blocking buffer (Odyssey Blocking Buffer 1:1 with PBS; Li-cor Biosciences; Lincoln, NE, USA), then in primary antibody overnight at 4°C using the following concentrations: Anti-GAPDH, 1:8000 (Imgenex, San Diego, CA); Anti-β-Tubulin, 1:4000 (Imgenex, San Diego, CA); Anti-Myelin Basic Protein (MBP), 1:4000 [AB62631] (Abcam, Cambridge, MA, USA). The blots were washed with PBS containing 0.05% Tween (Sigma, St. Louis, MO, USA) (3 × 10 min), and incubated for 1 h with the appropriate secondary antibody (1:8000 IRDye, Li-cor Biosciences, Lincoln, NE, USA). The blots were washed again in PBS-Tween (3 × 10 min) and bands were visualized using the Odyssey scanner (Li-cor Biosciences, Lincoln, NE, USA). The blots were stripped (Blot Restore Membrane Rejuvenation kit, Millipore, Billerica, MA, USA) and then reprobed with another primary antibody.

### Analyses

To quantify protein expression, blots were scanned using Odyssey Infrared Imaging System (Li-cor Biosciences, Lincoln, NE, USA) and the bands were quantified using densitometry (Li-cor Odyssey Software Version 3.0, Li-cor Biosciences, Lincoln, NE, USA). The density of each band was determined by subtracting the background, integrating the pixel intensity of the band, and dividing that intensity by the width of the band to control for variations in lane width. GAPDH was used as the loading control in this study after determining that GAPDH expression was not affected by either age or length of post-mortem interval (PMI, see Results). The expression of Classic- and Golli-MBP in each lane of the blot was divided by GAPDH expression in the same lane. To compare protein expression levels across blots, a control sample (mixture of all samples) was run on every gel, and for each sample on a blot the density was normalized to the density of the control sample. Finally, for each MBP protein, expression levels across runs were normalized using the average expression of the protein.

To analyze the developmental changes in Classic–and Golli-MBP we began by plotting scatterplots, showing the results from every run (gray dots) as well as the average for each sample (black dots), and histograms binning all the data into different age groups. We applied a model-fitting approach (Christopoulos and Lew, 2000) to determine the best fitting curve to the data presented in the scatterplots (gray dots) using Matlab. Significant curve fits are plotted on the scatterplots. We found that quadratic functions were good fits to each data set (y =PeakExp + A^*^(x - PeakAge)^2) and the best fitting curves were determined by least squares giving the goodness-of-fit (R), statistical significance of the fit (p), and 95% confidence interval (CI) for the age at the peak of the function. The age when mature levels of protein expression were reached was calculated from the parameters of the curve-fits and was defined as the age when protein expression first reached 90% of peak expression.

We compared differences among developmental stages by binning the data into the following age groups: neonatal (<0.4 years), infants (0.4–1 years), young children (1–4 years), older children (5–11 years), teens (12–20 years), young adults (21–55 years), and older adults (>55 years). Histograms were plotted using the mean and standard error of the mean (SEM) for each age bin. Statistical comparisons among age groups were made using an analysis of variance (ANOVA) and when significant (*p* < 0.05), Tukey's *post-hoc* comparisons were done to determine which age groups were significantly different.

We quantified changes in the relative expression of Classic–to Golli-MBP by calculating an index using the formula (Classic-MBP - Golli-MBP)/(Classic-MBP + Golli-MBP). The two families of MBP are genetically and functionally related so this index provides information about their combined development. The index can vary from −1 to +1 and this type of contrast index is commonly used in signal processes to reduce noise. Here we applied the index to analyze the balance between Classic- and Golli-MBP across the lifespan, where negative values represent more Golli-MBP and positive values more Classic-MBP. The index was plotted as described above. Briefly, a quadratic function was fit to all the index data and an analysis of variance was done to compare changes in the index among age bins.

In a previous study, we identified stages in cortical development when there was high inter-individual variability in the expression of various synaptic proteins (Pinto et al., 2015). We applied the same approach in this study to examine changes in inter-individual variability in expression of Classic- and Golli-MBP by calculating the Fano Factor (Variance-to-Mean Ratio, VMR) for each MBP protein. The VMR at each age was calculated using the average protein expression for each case and determining the mean and variance in expression within a window that included the case and the two adjacent ages. The VMRs for Classic- and Golli-MBP were plotted as scatterplots to visualize whether there are ages in development with high inter-individual variability.

## Results

### Loading control and postmortem interval

**Figure 1:**
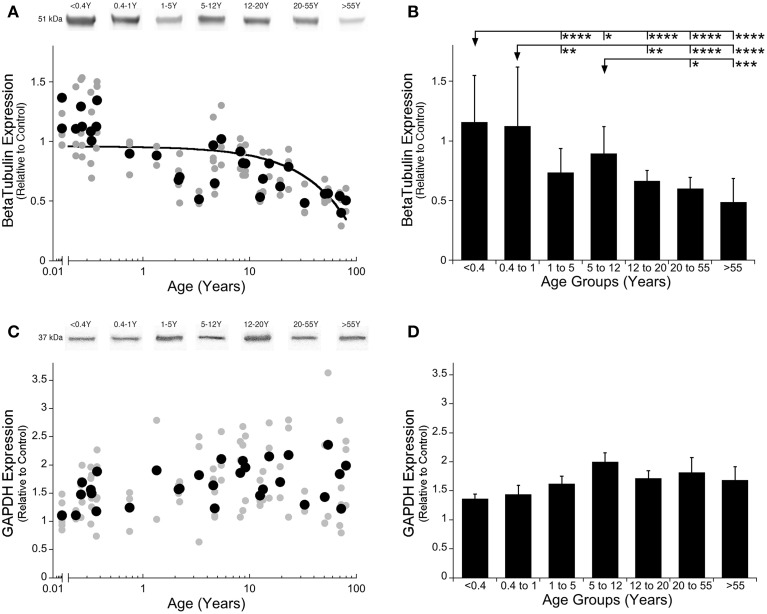
**β-Tubulin and GAPDH expression levels in human primary visual cortex across the lifespan**. Expression levels of β-Tubulin **(A,B)** and GAPDH **(C,D)** are relative to control, and plotted in scatterplots and age-grouped histograms. In the scatterplots **(A,C)**, gray symbols indicate values from individual runs, black symbols indicate the average between runs. Age is plotted on a logarithmic scale. Example bands from representative Western blots are pictured above. In the histograms **(B,D)**, developmental age group means and standard error for each group are plotted. **(A)** A quadratic function was fit to all β-Tubulin data points across the lifespan (*R* = 0.567, *p* < 0.0001). **(B)** Significant differences among age groups are identified (ANOVA, *F* = 18.715, *p* < 0.0001), and Tukey's post-hoc comparisons were made between groups (^*^p < 0.05, ^**^p < 0.01, ^***^p < 0.001, ^****^p < 0.0001). **(C)** There was no significant change in GAPDH expression across the lifespan. **(D)** There were no significant differences in expression of GAPDH among the developmental age groups.

Our first step was to determine the best loading control for this study by quantifying expression of the two most commonly used loading controls, GAPDH and β-Tubulin. We were looking for a loading control that had similar expression in human visual cortex across all ages. We found that β-Tubulin declined across the lifespan (Figure 1A; *R* = 0.5669, *p* < 0.0001; Figure 1B; ANOVA, *F* = 1 8.71, *p* < 0.0001). In contrast, GAPDH expression did not change across the lifespan as neither the ANOVA or curve-fits were significant (Figures 1C,D), therefore we selected GAPDH to use as the loading control. Next, we compared GAPDH expression with PMI and found no effect of the length of PMI on GAPDH expression (*R* = 0.05, n.s.). Finally, we assessed the effect of PMI on expression of Classic- and Golli-MBP to determine if any samples needed to be removed because the PMI was too long. We found no correlation with PMI for either myelin protein (Classic: *R* = 0.307, n.s.; Golli: *R* = 0.143, n.s.) so all samples were included in the study.

### Developmental of classic- and Golli-MBP

**Figure 2:**
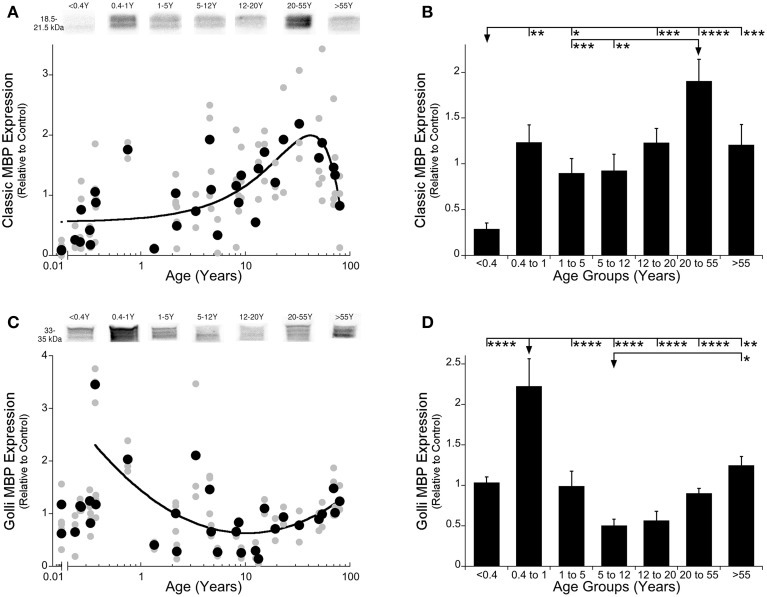
**Classic- and Golli-MBP expression levels in human primary visual cortex across the lifespan**. Expression levels of Classic-MBP **(A,B)** and Golli-MBP **(C,D)** are relative to control, and plotted in scatterplots and age-grouped histograms. In the scatterplots **(A,C)**, gray symbols indicate values from individual runs, black symbols indicate the average between runs. Age is plotted on a logarithmic scale. Example bands from representative Western blots are pictured above. In the histograms **(B,D)**, developmental age group means and standard error for each group are plotted. **(A)** A quadratic function was fit to all Classic-MBP data points across the lifespan (*R* = 0.59, *p* < 0.0001). A peak in expression of Classic-MBP was reached at 42 years of age (peak = 42 years 95% CI +/= 4.26 years). **(B)** Significant differences among age groups are identified (ANOVA, *F* = 10.248, *p* < 0.0001), and Tukey's post-hoc comparisons were made between groups (* p < 0.05, ** p < 0.01, *** p < 0.001, **** p < 0.0001). **(C)** An inverse quadratic function was fit to all the Golli-MBP data points older than 0.4 years of age (*R* = 0.61; *p* < 0.0001). The minimum expression was reached at about 10 years of age (minimum = 10.3 years 95% CI ± 3.8 years). **(D)** Significant differences among age groups are identified (ANOVA, *F* = 10.955, *p* < 0.0001), and Tukey's post-hoc comparisons were made between groups (^*^p < 0.05, ^**^p < 0.01, ^***^p < 0.001, ^****^p < 0.0001).

To study Classic- and Golli-MBP expression in human visual cortex, we used two approaches: model fitting to describe the developmental trajectories, and age binning of the data to compare among stages of development. We found a gradual increase in expression of Classic-MBP that continued into adult years, before a roll off in aging. That trajectory was well fit by a quadratic function (y = 1.999−0.0008182^*^(x-42.01)^2; *R* = 0.59, *p* < 0.0001) showing that Classic-MBP rose through development, increasing about 4-fold to the peak of expression at 42 years (95% CI ± 4.26 years) (Figure 2A). We defined the age when Classic-MBP matures as the age when expression reached 90% of the peak. For Classic-MBP that occurred at about 38 years of age showing the very slow and prolonged development of this myelin protein in human visual cortex.

We compared expression of Classic-MBP across developmental stages and found significant differences among the various stages (ANOVA; *F* = 10.25; *p* < 0.0001) (Figure 2B). Classic-MBP expression in neonates (<0.4 years) was less than all of the other age groups (Figure 2B; *p*-values range 0.0001–0.01). There was a jump in expression between neonates and infants (0.4–1 year) (*p* < 0.01), another significant increase between children (5–12 years) and young adults (20–55 years) (*p* < 0.01), followed by a trend to decline into aging (>55 years) (*p* = 0.14). Together, the curve-fitting and developmental stage comparisons showed that Classic-MBP in human visual cortex had prolonged development and substantial increase in expression.

The developmental trajectory for Golli-MBP was very different from Classic-MBP. Golli-MBP expression was very low in neonates (<0.4 years) and increased abruptly in slightly older infants. This early postnatal jump in Golli-MBP could be seen in both the scatterplot and age binned histogram (Figures 2C,D). Because of this pattern, we chose to apply model fitting to analyze development for cases older than 0.4 years of age. Expression of Golli-MBP expression for cases older than 0.4 years was well fit by an inverse quadratic function (y = 0.6301 + 0.15^*^(log(x)-log(10.31))^2; *R* = 0.61; *p* < 0.0001) (Figure 2C). Golli-MBP expression dropped from high levels to reach a minimum at about 10 years of age (Figure 2C; minimum = 10.3 years 95% CI ± 3.8) and then increased through adults into aging. Analysis of the age binned groups found similar results (Figure 2D; ANOVA; *F* = 10.95, *p* < 0.0001). Golli-MBP expression in infants (0.4–1 year) was significantly higher than all of the other age groups (Figure 2D; *p*-values range < 0.0001–0.01). Children (5–12 years) had the lowest level of Golli-MBP followed by a 2-fold increase in expression into aging (*p* < 0.0001). Thus, the rate of development of Golli- was faster than Classic-MBP, the peaks were at different ages, Golli- decreased while Classic-MBP increased through childhood, then they flipped and Golli- increased while Classic-MBP decreased into aging.

### Balance between classic- and Golli-MBP

**Figure 3:**
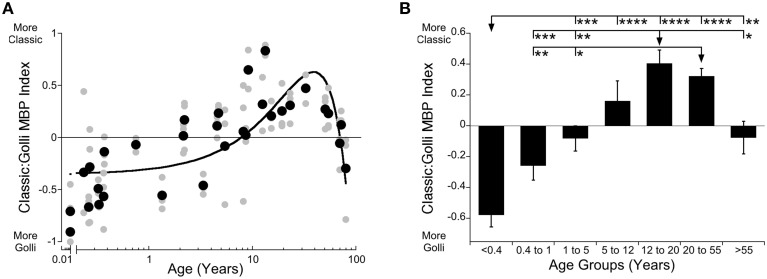
**Developmental changes of Classic- to Golli-MBP Index across the lifespan in human visual cortex**. Expression of the index results between Classic- and Golli-MBP are presented in a scatterplot **(A)**, where gray symbols indicate values from individual runs and black symbols indicate values indicate the average between runs. Age is plotted on a logarithmic scale. In a histogram **(B)**, developmental age group means and standard error of the index results in each group are plotted. **(A)** A quadratic function was fit to all the Classic:Golli index data points (*R* = 0.653, *p* < 0.0001), and shows a shift to relatively more Classic-MBP at 7.6 years, shows peak Classic-MBP expression at 38 years (38.3 years, 95% CI ± 2.7 years), and more Golli-MBP at 68 years of age. **(B)** Significant differences among age groups are identified (ANOVA, *F* = 16.35, *p* < 0.0001) and Tukey's post-hoc comparisons were made between groups (^*^p < 0.05, ^**^p < 0.01, ^***^p < 0.001, ^****^p < 0.0001).

Often changes in Classic- and Golli-MBP are described as moving in opposite directions. Even the current results, especially the histograms (Figures 2B,D) appeared to be complementary, but when we analyzed the relationship between Classic- and Golli-MBP we found no correlation (*R* = 0.177, n.s.), suggesting that there is no simple linear relationship for the amount of protein expressed by these two MBP families. However, there is a functional link between Golli- and Classic-MBP through calcium signaling (Paez et al., 2009; Smith et al., 2011), and in addition Golli-MBP KO mice have profound hypomyelination in the visual cortex (Jacobs et al., 2005). Those two findings provide evidence of a relationship, and so we analyzed the relative expression of Classic-to-Golli-MBP. We calculated an index [(Classic-MBP − Golli-MBP)/(Classic-MBP + Golli-MBP)] that could vary from −1 (only Golli-MBP) to +1 (only Classic-MBP) and provided information about how the balance between Classic- and Golli-MBP changed in human visual cortex across the lifespan. The scatterplot and age binned histogram of the Classic-to-Golli-MBP index showed gradual changes that continued across the entire lifespan (Figures 3A,B). A quadratic function was a good fit to the index results (y = 0.6308-0.0006492^*^(x-38.92)^2; *R* = 0.65, *p* < 0.0001) and captured the gradual shift from more Golli- to more Classic-MBP, then back to relatively more Golli-MBP in older adults (Figure 3A). The function crossed from more Golli- to more Classic-MBP at 7.6 years of age, reached a peak with more Classic-MBP at about 38 years of age (38.3 years, 95% CI ± 2.7 years) and crossed back to more Golli-MBP at about 68 years (Figure 3A). There were also significant differences among the developmental stages (Figure 3B; ANOVA, *F* = 16.35, *p* < 0.0001). Teens (12–20 years) and young adults (20–55 years) had relatively more Classic-MBP than the other age groups. The initial shift in the index was driven by the more rapid loss of Golli-MBP, then the switch to Classic-MBP by the prolonged increase in Classic-, and the change into aging by complementary decrease and increase in Classic- and Golli-MBP, respectively. These results suggest three phases of development, an early stage (<8 years) when Golli-MBP is changing, an intermediate stage (8–67 years) when Classic-MBP changes and a late stage (>68 years) when both families of MBP change.

### Inter-individual variability

**Figure 4:**
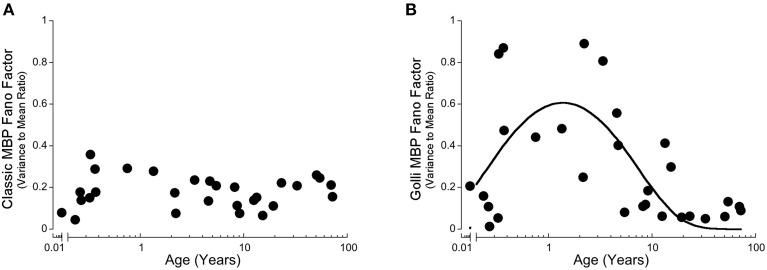
**Development of the Variance-to-Mean Ratio (VMR) for Classic-MBP (A) and Golli-MBP (B). (A)** Classic-MBP had no change in VMR across the lifespan. **(B)** A quadratic function was fit to all Golli-MBP VMR data points, and had a period of high VMR early on with a peak at about 1.4 years of age.

In our recent paper, we discovered stages in development of human visual cortex when there was a high degree of inter-individual variability in expression of synaptic proteins (Pinto et al., 2015). To assess if Classic- or Golli-MBP had similar waves of inter-individual variability we calculated the Fano factor (Variance-to-Mean Ratio, VMR) for a running window of three adjacent ages for each protein, and plotted the VMR across the lifespan. The VMR for Classic-MBP was low at all ages, suggesting that there was little inter-individual variability in expression of Classic-MBP in visual cortex (Figure 4A). In contrast, Golli-MBP had a period with higher inter-individual variability between about 0.4 and 5 years of age that peaked at 1.4 years (y = 0.920387^*^exp(−0.285089/x−0.151975^*^x) (Figure 4B). These findings highlight another way that expression of the two families of MBP differ and raises the possibility that the wave of high inter-individual variability for Golli-MBP reflects a period of vulnerability in development of human visual cortex.

## Discussion

The changes in Classic- and Golli-MBP found in this study of human visual cortex highlight the complex nature of myelin expression in the brain. We found that these two families of MBP follow different developmental trajectories, have three stages of a Classic:Golli balance, and only Golli-MBP has high inter-individual variability during childhood. We have three main conclusions from this study.

### Different developmental trajectories for classic- and Golli-MBP in human visual cortex

First, Classic- and Golli-MBP follow different trajectories during development and aging of human visual cortex. Classic-MBP had a prolonged period of development that extended well into adulthood and then declined into aging. The pattern of prolonged development is consistent with findings from anatomical and brain imaging studies of myelin in human cortex. Anatomical measurements of myelin sheath density from postmortem human cortex showed that maturation continued well into the third decade of life (Miller et al., 2012). Brain imaging of myelin content in human cortex also showed that gray matter myelin continued to increase into adulthood (Grydeland et al., 2013; Shafee et al., 2015). Together, those results confirm that myelin development in human visual cortex extends well beyond the window of time (6–10 years of age) that represents the end of the sensitive period for development of lazy-eye (amblyopia) (Epelbaum et al., 1993; Keech and Kutschke, 1995; Lewis and Maurer, 2005). Our study also identified that Classic-MBP expression continued to change in older adults by declining into aging. The loss of Classic-MBP into aging is similar to findings from *in vivo* imaging of cortical myelin (Grydeland et al., 2013) and may be related to degeneration of myelin sheath integrity in aging monkey visual cortex (Peters et al., 2008).

The development of Golli-MBP was different from Classic-MBP providing additional evidence that the various myelin proteins follow different developmental trajectories (Miller et al., 2012). We found an abrupt increase in Golli-MBP at about 4–6 months of age, a peak around 1 year followed by a decline to the minimum level at about 10 years, and then a gradual increase into aging. The abrupt increase in Golli-MBP corresponds with the start of the sensitive period for development of binocular vision (Banks et al., 1975) and matches the timing of rapid changes in the balances among pre- and post-synaptic proteins in human visual cortex (Pinto et al., 2015). Perhaps the coincidence in timing of abrupt changes in Golli-MBP and synaptic proteins helps to trigger the start of a period of heightened plasticity when visual experience readily sculpts maturation of circuits in the visual cortex.

It is interesting to consider how the early changes in Golli-MBP could contribute to mechanisms that facilitate and limit experience-dependent plasticity in the developing visual cortex. We chose to study Golli-MBP because it has been called a “molecular link” between the nervous and immune systems (Pribyl et al., 1993), and its functions during neural development are consistent with Golli-MBP contributing to sensitive period plasticity. Golli-MBP is found in developing oligodendrocytes (Campagnoni et al., 1993), neurons (Landry et al., 1996), T-cells (Feng et al., 2000, 2004) and macrophages (Papenfuss et al., 2007), and is a major regulatory protein for calcium influx into both oligodendrocytes (Paez et al., 2007) and T-cells (Feng et al., 2004). Its expression during early development increases cell migration, proliferation, extension, and retraction which contributes to maturation of neuroglia (Paez et al., 2009) and is consistent with supporting developmental plasticity. Golli-MBP is an intrinsically disordered protein that provides many sites for protein-protein interactions (Ahmed et al., 2007) that may be candidate sites to mediate experience-dependent plasticity.

At the other end of the lifespan, we found an age-related increase of Golli-MBP in visual cortex. Golli-MBP plays an important role in myelin repair, since Golli over-expressing mice have increased survival and proliferation of remyelinating oligodendrocytes (Paez et al., 2012). The age-related increase in Golli-MBP may reflect an increase in oligodendrocytes and changes in myelination such as thicker and shorter myelin segments that have been found in visual cortex of old monkeys (Peters et al., 2001, 2008; Peters and Sethares, 2004). Therefore, as a molecular link between the nervous and immune systems, Golli-MBP may bridge those systems and contribute to neuroimmune driven neurodegenerative diseases. Unfortunately, there have been no studies of Golli-MBP function in the aging brain and to the best of our knowledge our finding of increased Golli-MBP expression in human visual cortex is the first report of Golli-MBP in the aging brain. Clearly, more studies are needed to determine how Golli-MBP functions in the aging cortex and what role it plays in neuroimmune processes involved in neurodegeneration.

### Three stages of MBP development

Our second conclusion is that the balance between Classic- and Golli-MBP in human visual cortex has three stages. We found an early stage during childhood (<8 years) when Golli-MBP dominated, an intermediate stage (8–67 years) when there was more Classic-MBP, and a late stage (>68 years) that shifted back to more Golli-MBP. The three stages were driven by the different trajectories for Classic- and Golli-MBP. The early stage was marked by rapid changes in Golli-MBP expression while the intermediate stage had relatively constant Golli- and gradually increasing Classic-MBP. Finally, the aging stage reflected both an increase of Golli- and loss of Classic-MBP. Examining the balance between Classic- and Golli-MBP is a new way to study MBP changes across the lifespan and although these proteins do not interact directly, Golli-MBP can affect expression of Classic-MBP. For example, over-expression of Golli-MBP significantly delays expression of Classic-MBP and the process of myelination, (Jacobs et al., 2009) while knocking out Golli causes permanent hypomyelination of the visual cortex (Jacobs et al., 2005). Perhaps, higher expression of Golli-MBP during development of human visual cortex delays myelination, thereby holding off one of the brakes on critical period plasticity (McGee et al., 2005).

The timing of the shift to more Classic-MBP corresponds with the end of the sensitive period for development of amblyopia in children (Epelbaum et al., 1993; Keech and Kutschke, 1995; Lewis and Maurer, 2005). That stage of the Classic-:Golli-MBP balance may contribute to a period of stability in the visual cortex as Classic-MBP expression increases into adulthood. Traditionally, myelination is viewed as supporting signal transduction by enabling saltatory propagation of neural impulses. More recently, myelin has been shown to be important for supporting (Fields, 2005, 2014) or limiting synaptic plasticity (McGee et al., 2005), and transporting metabolites to axons (Saab et al., 2013; Nave and Werner, 2014). Glutamate release promotes formation of the myelin sheath around axons and increases synthesis of Classic-MBP thereby linking myelination with electrically active axons (Wake et al., 2011). In our recent study of synaptic proteins in human visual cortex (Pinto et al., 2015), we found that the glutamate receptor scaffolding protein, PSD-95, increases during late childhood at a similar point in development when Classic-MBP expression takes off. Interestingly, Classic-MBP has an SH3 ligand that can bind to the SH3 binding site on PSD-95 (Polverini et al., 2008), thereby providing a mechanism where Classic-MBP may contribute to SH3-mediated establishment of a stable lattice of PSD-95 molecules at the post-synaptic density (Sturgill et al., 2009). Myelin also functions to supply axons with metabolites and neurotrophic factors that are necessary to maintain healthy neural connections (Nave and Werner, 2014). These two lines of evidence suggest that the stage when Classic-MBP dominates is driven by an increase in excitatory activity and supports a period of optimal axonal energy metabolism.

The late stage of the Classic-:Golli-MBP balance was the shift to relatively more Golli-MBP. There have been no studies of Golli-MBP function in the aging cortex, but its roles in demyelination and remyelination (Paez et al., 2012), and multiple sites for protein-protein interactions make it an ideal candidate for regulating changes to myelination in the aging cortex. In addition, this loss of Classic-MBP suggests that one component of impaired circuit function in the aging cortex may be reduced metabolic support for axons.

### A wave of Golli-MBP inter-individual variability during childhood

Our third conclusion is that Golli-MBP goes through a period of high inter-individual variability in childhood. In our previous study of synaptic protein development in human visual cortex, we found protein specific waves of high inter-individual variability throughout childhood (Pinto et al., 2015). In the current study, only Golli-MBP had a wave of high inter-individual variability in childhood, while Classic-MBP had low variability across the lifespan. The timing of the wave of Golli-MBP variability was similar to the wave for PSD-95 (Pinto et al., 2015) with both being low in neonates, high during infancy and childhood, then dropping to low levels in adults. The difference in variability between Golli- and Classic-MBP suggests that Golli-MBP expression is more dynamic than myelin structural proteins such as Classic-MBP. Since Golli-MBP is involved in signaling between neurons, oligodendrocytes, and immune cells, the wave of variability in childhood is likely a complex interaction reflecting a period of vulnerability between neural and immune systems. Perhaps it is linked to the high risk of infection during childhood. In contrast, we were surprised by the lack of inter-individual variability for Golli-MBP in aging, especially since neuroimmune regulation is impaired in age-related neurodegeneration (Frank-Cannon et al., 2009).

### Studying myelin in visual cortex

Myelin proteins bind to Nogo and PirB receptors to inhibit axon regeneration (Hu and Strittmatter, 2004; Atwal et al., 2008), and in the visual cortex both of those receptors limit experience-dependent plasticity (McGee and Strittmatter, 2003; Syken et al., 2006). Mutations to or blocking either Nogo-66 or PirB receptors allow ocular dominance plasticity to continue into adulthood (McGee et al., 2005; Syken et al., 2006; Bochner et al., 2014) showing a link between myelin proteins, neuroimmune processes, and visual experience-dependent plasticity. Over-expression of Golli-MBP significantly delays myelination (Jacobs et al., 2009) while knocking out Golli-MBP causes profound hypomyelination that is restricted to the visual cortex (Jacobs et al., 2005). Thus, both too much or no Golli-MBP affect myelination. The specific effect of knocking out Golli-MBP on myelination of the visual cortex suggests that it plays a special role in maturation of that cortical area.

In this study, we found that the shift from more Golli- to more Classic-MBP in human visual cortex coincides with the end of the sensitive period for development of amblyopia. Futhermore, MBP mRNA is reduced in the visual cortex of patients with schizophrenia (Matthews et al., 2012) suggesting that it may contribute to the pathophysiology underlying changes in their visual perception. Thus, the visual cortex is an interesting cortical area for studying the role of myelin proteins in disease.

Previous studies of myelin in the human brain have focused on white matter maturation (e.g., Paus, 2010; Guleria and Kelly, 2014) and diseases related to changes in the white matter (Fields, 2008). In contrast, our study quantified Classic- and Golli-MBP expression in human visual cortex. The findings are timely because novel MRI techniques are driving renewed interest in the role of myelin function in human cortex (Glasser et al., 2014). Furthermore, the changes in Golli-MBP expression in human visual cortex raise new questions about its role as a molecular link between neural and immune systems at different stages of the lifespan.

### Conflict of interest statement

The authors declare that the research was conducted in the absence of any commercial or financial relationships that could be construed as a potential conflict of interest.
